# Nanomaterial-Based SPR Sensing Chip for Detection of Metal Ion Mixtures in Aquatic Environment

**DOI:** 10.3390/mi17091067

**Published:** 2026-09-09

**Authors:** Jie Li, Xiaomeng Zhang, Chong Yue, Wenbin Yin

**Affiliations:** 1 Institute of Urban Underground Space and Energy, The Chinese University of Hong Kong, Shenzhen 518172, China; russellli@cuhk.edu.cn (J.L.); zhangxiaomeng@cuhk.edu.cn (X.Z.); yinwenbin@cuhk.edu.cn (W.Y.); 2 Chongqing Academy of Metrology and Quality Inspection, Chongqing 401123, China; 3 College of Optoelectronic Engineering, Chongqing University, Chongqing 400030, China

**Keywords:** SPR, sensor, metal ion mixtures, nanomaterial, aquatic environment

## Abstract

Aquatic heavy metal pollution poses substantial risks to ecological systems and human health, attributable to the toxic properties and bioaccumulative behaviors of metallic ions, including Hg[II], Zn[II], and ion mixtures. A novel SPR sensing chip based on a Ag-BiFeO_3_-MoS_2_–graphene hybrid structure is proposed and analyzed for detection of metal ion mixtures. Systematic tuning is implemented for Ag and BiFeO_3_ film thicknesses to boost the overall sensing capability, aiming to acquire minimized reflectance together with favorable detection sensitivity. Following this step, we investigate how different quantities of MoS_2_ and graphene layers affect the sensing properties of the SPR biosensor. Analytical outcomes demonstrate that configurations adopting monolayer MoS_2_ and graphene achieve the maximum phase sensitivity. Moreover, the optimized SPR chip architecture achieves sensing sensitivity two orders of magnitude greater than conventional sensor setups. Numerical investigations are further carried out to evaluate the sensor’s response toward various heavy metal ion species. The maximal sensitivity of 1.599 × 10^6^ deg/RIU is realized when detecting Zn[II]. Under this optimal structural setup, the spatial electric field distributions responding to variations in the refractive index of the sensing medium are also characterized. The remarkable sensitivity of the presented sensor configuration makes it a more competitive choice for deployment in further biological detection scenarios.

## 1. Introduction

Mercury (Hg), zinc (Zn) and mixed ions represent highly hazardous environmental heavy metal contaminants capable of accumulating inside living organisms through bio-logical enrichment. Once accumulation exceeds the body’s tolerance threshold, chronic toxic effects occur, making this field a long-term hotspot in environmental science and public health [1,2,3]. In aquatic systems, these metal ions undergo biomagnification through the food chain, causing widespread toxic harm to aquatic ecosystems, and have become a key interdisciplinary topic in environmental chemistry, ecotoxicology and health science [4,5,6,7]. Hg, with high diffusivity and lipophilicity, easily penetrates biological membranes, oxidizes to divalent ions in blood, and accumulates in brain and kidney tissues to induce organic damage [8,9]. Excessive Zn intake causes acute irritation of the gastrointestinal tract, and chronic exposure disrupts trace element metabolism and impairs liver and kidney functions [10,11]. In this respect, developing precise detection techniques for heavy metal ions is of great practical significance.

SPR sensors are capable of detecting slight variations in refractive index of analyte media, thus making them applicable to various applications such as bio-chemical analysis [12], health status monitoring [13], environmental monitoring [14] and many others. Based on this sensing mechanism, a new heavy metal ion detection platform based on SPR has been fabricated to target ions such as Hg[II], Zn[II] and mixed ions, and for use in analyzing heavy metal contaminants in aqueous systems. Compared with conventional analytical techniques, the proposed scheme possesses prominent merits, including outstanding sensitivity, real-time measurement capacity, simple operating procedures, label-free detection, strong anti-interference capability, and fast response speed [15,16,17]. The prism–metal–dielectric stacked Kretschmann framework represents the most established strategy to stimulate surface plasmon resonance [18]. A Ag thin film is selected as the metallic layer on account of its superior detection precision. However, traditional silver-based SPR biosensors generally suffer from inherently limited detection sensitivity.

Exploiting their particular optoelectronic properties, two-dimensional materials such as graphene and transition metal dichalcogenides (TMDCs) are regarded as promising overlay materials for the performance enhancement of SPR sensors [19]. Their high real dielectric constant improves light absorption by the metal layer [20], and their large surface-to-volume ratio and tunable biocompatibility enable sensitivity enhancement for biosensing applications [21]. Such coatings also provide anti-oxidation protection to the underlying metal film [22,23]. Numerous 2D material-modified SPR sensors have thus been proposed and studied. Graphene has been explored for sensitivity enhancement [24], and Zeng et al. constructed a high-sensitivity graphene–MoS_2_ hybrid SPR biosensor for this purpose [25]. Previous research [26] explored the sensitivity improvement brought by integrating graphene with an air spacer layer. Meanwhile, other TMDC materials, including WS_2_, MoS_2_ and WSe_2_, were combined with silicon substrates to achieve enhanced sensing performance [27]. Inserting auxiliary thin films, including silicon, BaTiO_3_ and TiO_2_, between metallic films and graphene represents another feasible optimization strategy for advancing the overall sensitivity of SPR sensing devices.

As a representative perovskite-structured multiferroic oxide, bismuth ferrite (BiFeO_3_, BFO) crystallizes within the R3c space group (lattice parameters: a = 3.958 Å; α = 89.30°). Its ferroelectric and magnetic characteristics have been extensively explored in existing studies [28,29,30,31]. This material features an adjustable bandgap between 2.09 eV and 2.32 eV, allowing carriers to be effectively excited upon visible light illumination. In addition, BFO exhibits outstanding chemical stability, an intrinsic polarization field, and a work function equal to 4.7 eV [32,33,34]. Nanostructured BFO exhibits improved electrical conductivity and enlarged specific surface area originating from low-dimensional quantum confinement effects, which facilitates sufficient, stable interfacial contact with 2D materials such as graphene [35,36,37]. Owing to its multiferroic characteristics, lead-free nontoxic nature and high Neel temperature, BFO has broad applications in spintronics and ultrafast opto-electronics and shows great promise in high-performance magnetic and gas sensing [38,39,40,41,42,43]. In particular, its nearly zero imaginary dielectric constant in the visible band enables ultra-low-loss polariton transmission [44]. In the present passive and unbiased transfer-matrix model, however, the multiferroic character, work function, and bandgap are not introduced as independent fitting parameters. The calculated enhancement is governed directly by the BFO complex’s permittivity and thickness. The other material descriptors are relevant mainly to interfacial charge transfer, optical loss, film quality, and possible future electro-optic or magneto-optic tuning.

Targeting the requirement of heavy metal ion monitoring within aqueous environments, this work designs an SPR sensor adopting Ag-BFO-MoS_2_–graphene hybrid architecture for the improvement of phase sensitivity. Through systematic optimization of the MoS_2_ and graphene layer number, and the thickness of the Ag and BFO, the comprehensive performance of the sensing device can be significantly improved. This SPR sensing platform can be extended to disease biomarker detection and gas monitoring scenarios alongside its primary use in aqueous heavy metal ion detection. All numerical computation work in this study is implemented via MATLAB 2021b software, and the corresponding simulation conditions and parameter settings are detailed later in this paper.

## 2. Theory and Design Methodology

**Figure 1:**
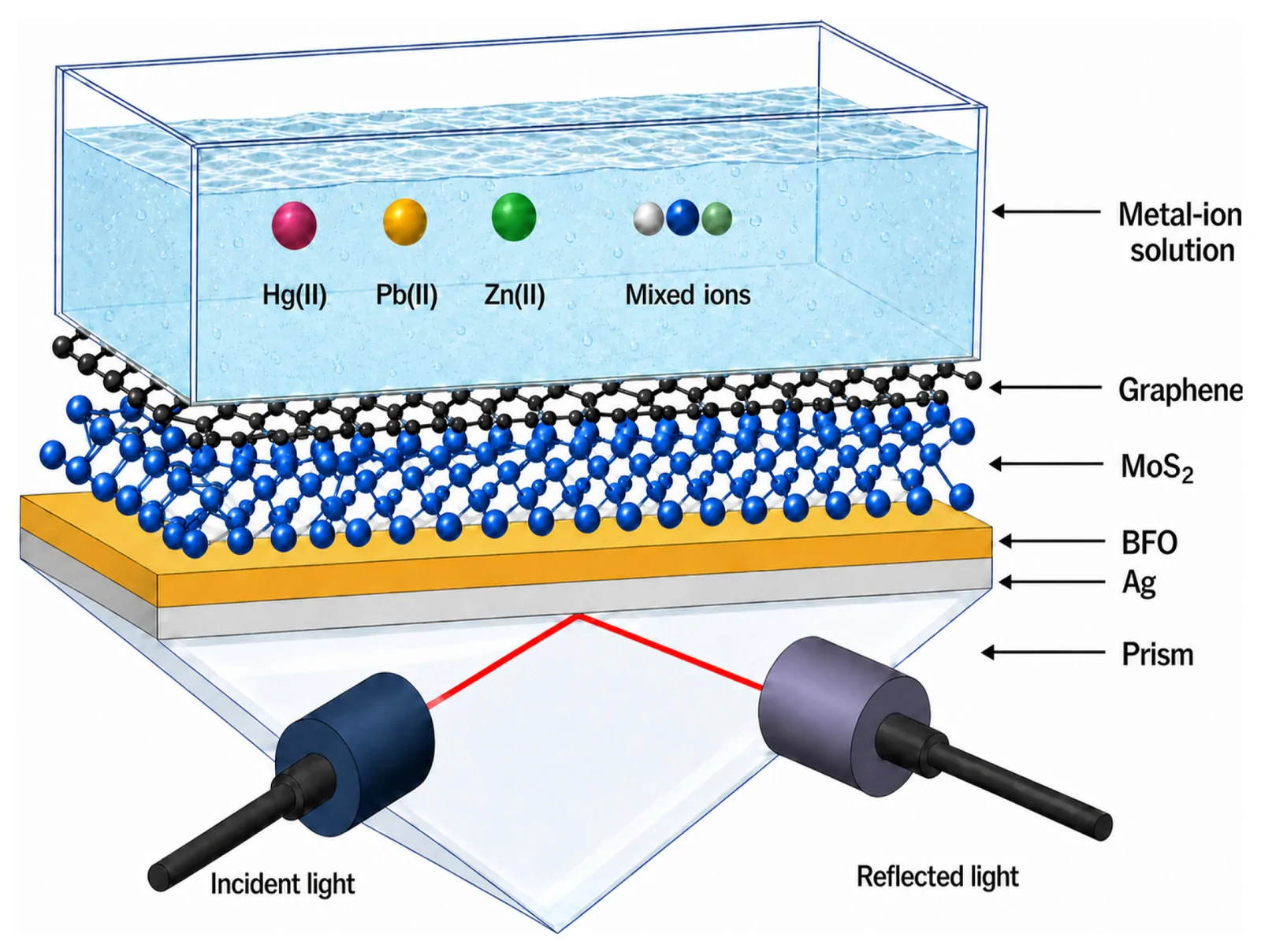
Structural schematic of phase-sensitive SPR system applied to mixed metal ion detection in water.

**Figure 2:**
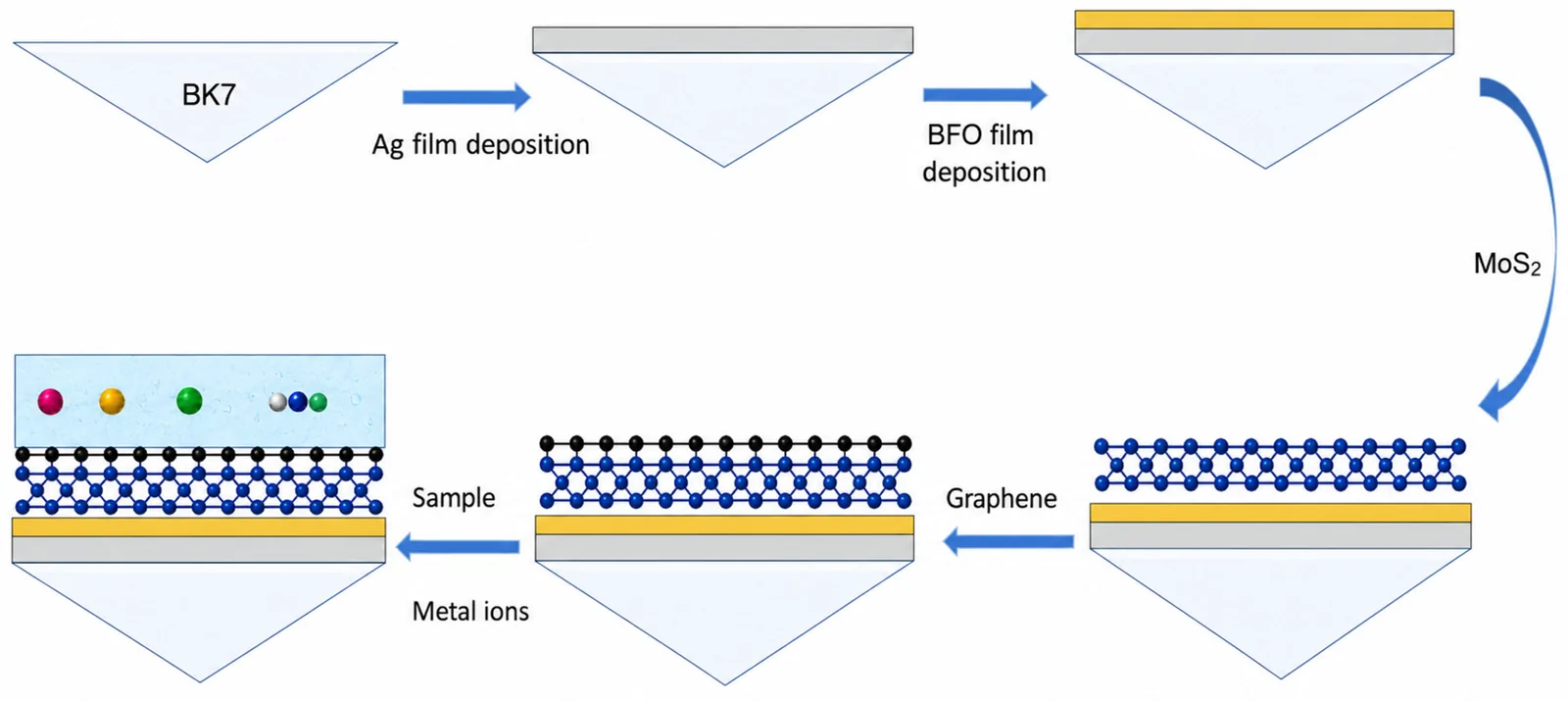
Construction process of SPR sensing structure.

A schematic layout of a phase-sensitive SPR system intended for metal ion mixtures in an aquatic environment is illustrated in Figure 1. The devised biosensor is built with six functional layers, namely, BK7 (n_Bk7_ $n_{BK7}=1.5151$), Ag ($n_{Ag}=0.0502+4.2347i$), BFO ($n_{BFO}=2.711$), MoS_2_, graphene, and the sensing medium. The characteristic optical parameters of all 2D materials adopted in this work at 633 nm are summarized in Table 1. The sensing medium is divided into four groups, composed of Hg[II], Zn[II], Hg[II]/Pb[II] and Hg[II]/Pb[II]/Ni[II]/Zn[II]/Cu[II]. Refractive index data for each of the three media are listed in detail in Table 2. Figure 2 schematically illustrates the layer-by-layer deposition scheme implemented on the prism substrate.

In this work, the transfer matrix method (TMM) coupled with the Fresnel equations is adopted to numerically compute SPR response curves with Equations (1)–(4), and further utilized to evaluate the reflectance properties, phase variation, and phase sensitivity of the proposed sensor.

(1) $$M=\prod_{m=1}^{N}M_{m}=\left[\begin{array}{cc} \cos\beta_{m} & -\frac{i}{q_{m}}\sin\beta_{m}\\ -q_{m}\sin\beta_{m} & \cos\beta_{m} \end{array}\right]$$

(2) $$\left\{\begin{array}{l} q_{k}=\frac{{\left(\varepsilon_{k}-n_{0}^{2}\sin^{2}\theta_{0}\right)}^{\frac{1}{2}}}{\varepsilon_{k}}\;\;\;\;TM\text{-}wave\\ q_{k}={\left(\varepsilon_{k}-n_{0}^{2}\sin^{2}\theta_{0}\right)}^{\frac{1}{2}}\;\;\;TE\text{-}wave \end{array}\right.$$

(3) $$\beta_{m}=\frac{2\pi d_{m}}{\lambda }{\left(\varepsilon_{m}-n_{0}^{2}\sin^{2}\theta_{0}\right)}^{\frac{1}{2}}$$

The terms $\beta_{m}$ and $q_{k}$ correspond to the phase factor and optical admittance, respectively. $\varepsilon_{m}$ and $\theta_{0}$ stand for the dielectric permittivity and incident angle. The reflection coefficient, represented by the symbol r, can be calculated for both transverse magnetic (TM) and transverse electric (TE) polarizations with the expression presented below:

(4) $$r=\frac{\left(M_{11}+M_{12}q_{N})q_{0}-(M_{21}+M_{22}q_{N}\right)}{\left(M_{11}+M_{12}q_{N})q_{0}+(M_{21}+M_{22}q_{N}\right)}$$

The TM-waves reflectivity, TM-waves phase, TE-waves phase and phase sensitivity can be written as follows:

(5) $$R_{TM}={\left|r_{TM}\right|}^{2}$$

(6) $$\phi_{TM(TE)}=\arg(r_{TM(TE)})$$

(7) $$\phi_{d}=\left|\phi_{TM}-\phi_{TE}\right|$$

(8) $$S=\frac{\Delta \phi_{d}}{\Delta n_{\mathrm{ion}}}$$

## 3. Results and Discussion

After defining the refractive index (RI) values of all functional layers within the proposed sensor structure, the thickness optimization of silver and BFO thin films is initially carried out on the basis of a baseline configuration containing monolayer MoS_2_ and graphene simultaneously. To guide this optimization, two parametric curves are constructed to characterize how film thickness correlates with two key performance indicators: sensitivity (S) and minimum reflectance (R_min_). The optimal thickness combination is identified by the criterion that sensitivity is maximized while R_min_ is kept at the lowest attainable level. Using these optimized thickness parameters, reflectance is simulated and compared across multiple structural configurations, including the proposed sensing architecture. Subsequently, the impacts of stacking additional MoS_2_ and graphene monolayer on both sensitivity and R_min_ are systematically investigated. Finally, the dependence of sensitivity and R_min_ on the RI of the sensing medium is demonstrated.

### 3.1. Thickness Optimization of Layers

**Figure 3:**
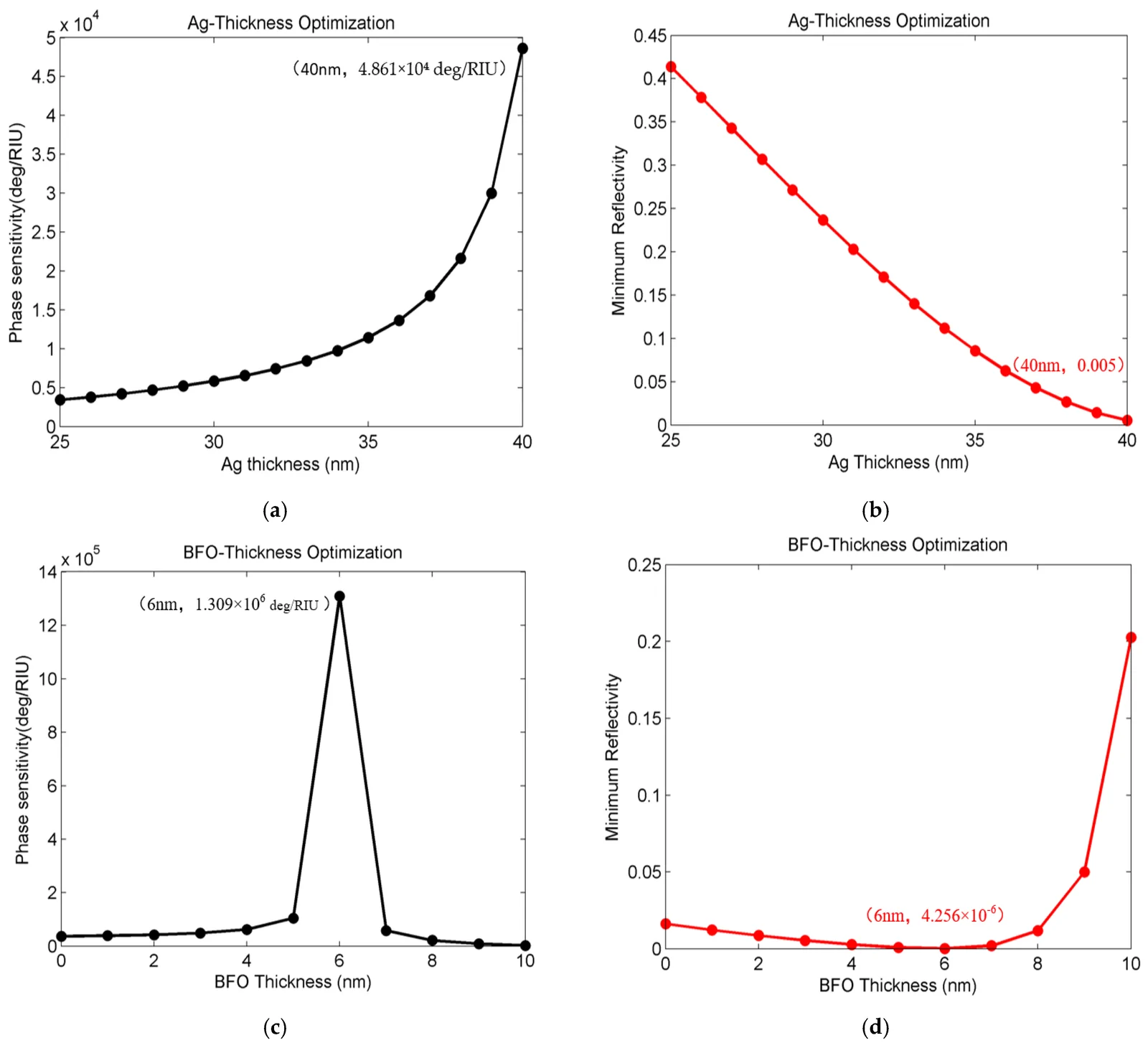
Thickness parameter optimization for Ag and BFO film using phase sensitivity and minimum reflectance as evaluation metrics under a monolayer MoS_2_–graphene baseline configuration: (**a**) Ag thickness–phase sensitivity; (**b**) Ag thickness–minimum reflectivity; (**c**) BFO thickness–phase sensitivity; (**d**) BFO thickness–minimum reflectivity.

Using Hg[II] as an example for detection, Figure 3 depicts how sensitivity and minimum reflectance (R_min_) evolve as the thicknesses of the Ag and BFO thin films are individually adjusted. All functional layers are incorporated into the numerical simulation, and a sequential single-parameter optimization routine is applied: for each round of calculation, only one layer’s thickness is varied; meanwhile, other layered components maintain typical baseline dimensions: the silver thin film is fixed at 40 nm, while the BFO layer adopts a thickness of 3 nm. Concretely, the Ag layer thickness is first optimized against both sensitivity and R_min_, with the BFO layer maintained at their baseline values of 3 nm. Subsequently, the BFO layer thickness is tuned using the already determined optimal Ag thickness. The full thickness optimization workflow is carried out under the configuration where both MoS_2_ and graphene exist as a single monolayer. Figure 3 reveals that sensor sensitivity rises monotonically as the silver layer thickness varies from 25 nm to 40 nm. The sensitivity value increases from 3.434 × 10^3^ deg/RIU up to 4.861 × 10^4^ deg/RIU within this scanning interval. Over the same thickness interval, the minimum reflectance (R_min_) varies between 0.413 (at 25 nm) and 0.005 (at 40 nm), and the lowest R_min_ value of 0.005 is achieved at a Ag thickness of 40 nm. To strike a balance between achieving the minimum reflectivity (R_min_) and appropriate device geometrical parameters, 40 nm is determined as the optimal thickness of the silver functional layer. As a step in the layer-by-layer optimization routine, the BFO thin film thickness is systematically swept from 0 nm to 10 nm to assess its impact on sensing performance. The sensor delivers a peak sensitivity of 1.309 × 10^6^ deg/RIU at a BFO thickness of 6 nm, and this figure declines steadily to 2.109 × 10^3^ deg/RIU when the film thickness increases to 10 nm. In terms of minimum reflectance (R_min_), its lowest magnitude of 4.256 × 10^−6^ is observed at a BFO layer thickness of 6 nm. Balancing the dual objectives of minimizing R_min_ and maintaining a favorable level of sensing sensitivity, a thickness of 6 nm is confirmed as the optimum structural parameter for the BFO thin film. Taken together, the stepwise layer-by-layer optimization workflow yields the final set of optimal thickness parameters: 40 nm for the Ag layer and 6 nm for the BFO thin film.

Sequential single-parameter scanning is a clear and efficient approach for evaluating the effect of each thin-film layer. It is particularly useful for preliminary screening within a realistic thickness range. However, this method is essentially a coordinate search and cannot prove that the result is globally optimal. The Ag and BFO layers together affect optical impedance matching, light absorption, and surface plasmon coupling. Their influences are therefore likely to interact. The final thickness selection may vary with the initial settings, scanning order, and step size. Other combinations that are not covered by the two one-dimensional scans may offer a better balance between phase sensitivity and (R_min_). A full two-dimensional scan of the Ag and BFO thicknesses would provide the most direct validation. It might show nearby solutions or other Pareto-efficient combinations. The proposed approach could give different results for a non-convex response surface or for a problem with additional variables and fabrication constraints. For these cases, Bayesian optimization, particle swarm optimization and genetic algorithms could be considered. Nevertheless, for the present bounded two-variable problem, a sufficiently fine two-dimensional grid is more transparent and reproducible. Within the tested range, the 6 nm BFO layer provides a high maximum sensitivity, while the response to Ag thickness follows a monotonic trend. The 40 nm Ag and 6 nm BFO structure is therefore identified as the preferred operating point based on the current sequential scans. It should not be regarded as a mathematically proven global optimum.

### 3.2. Influences of Graphene and MoS_2_ Stacking Layers

**Figure 4:**
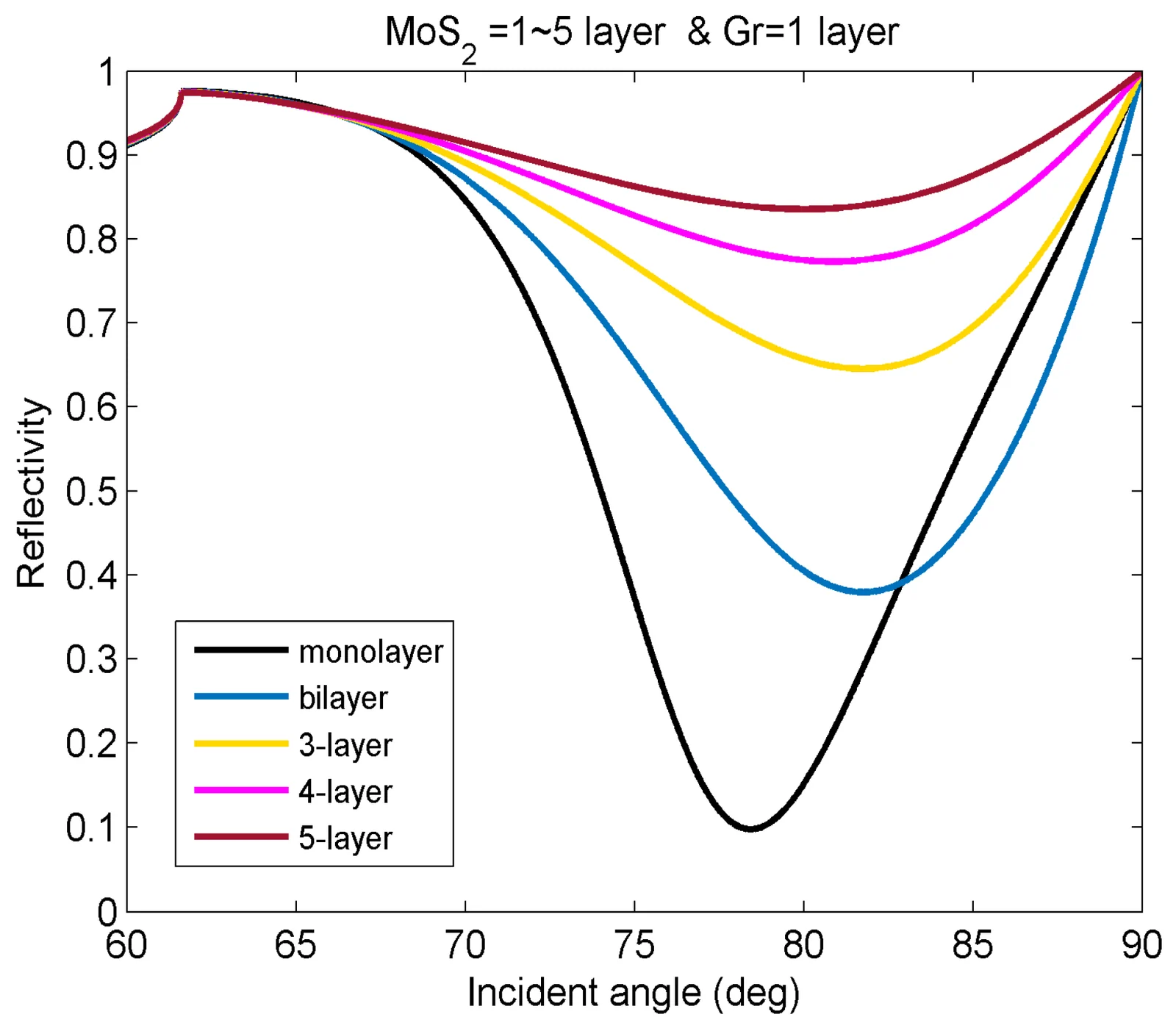
Reflectivity evolution as a function of stacked MoS_2_ counts under preoptimized Ag and BFO film thicknesses with a monolayer graphene baseline configuration.

**Figure 5:**
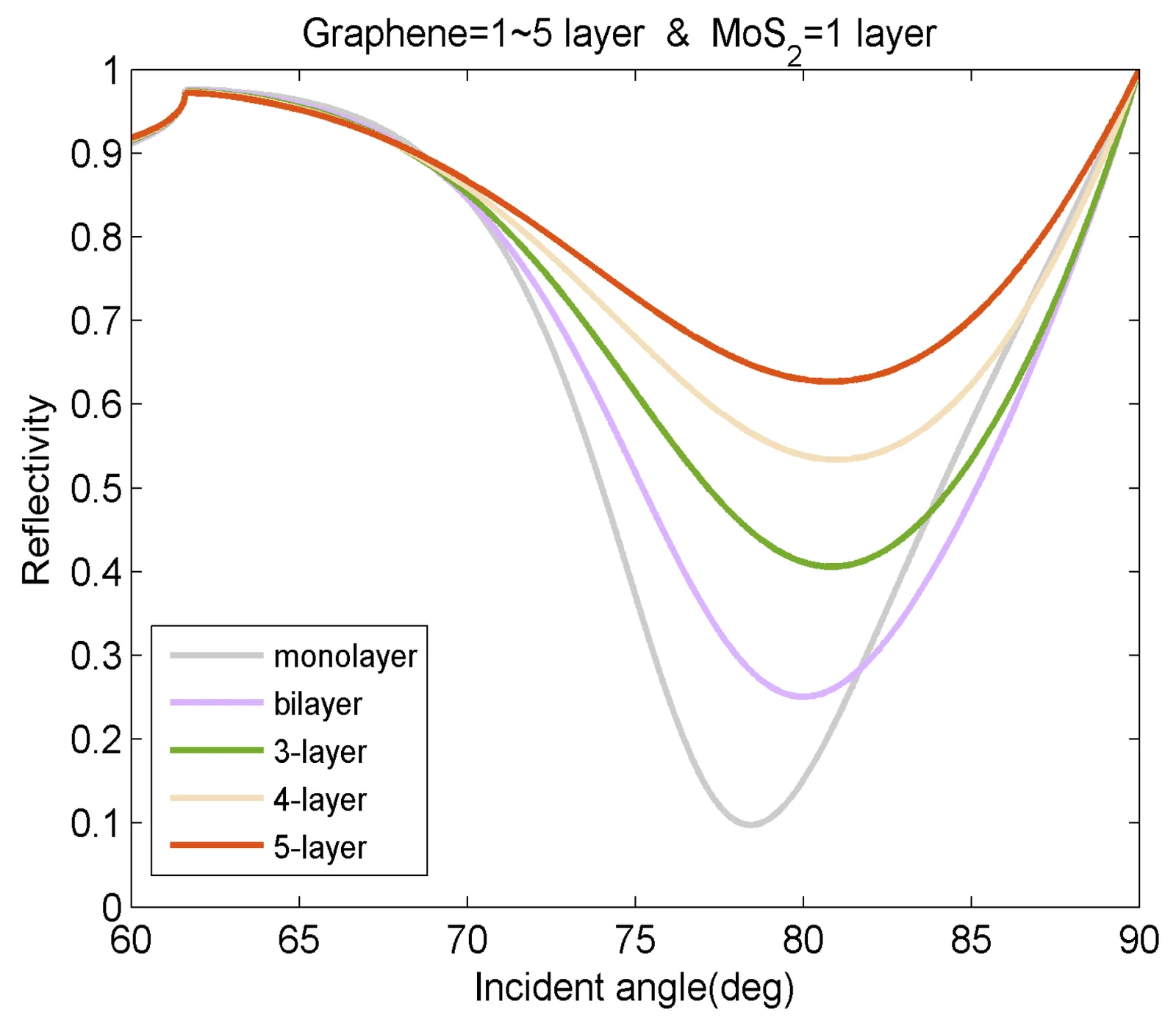
Reflectivity evolution as a function of stacked graphene layer counts under preoptimized Ag, BFO film thicknesses and monolayer MoS_2_ baseline configuration.

Reflectance obtained under the optimized thicknesses of Ag and BFO films are displayed in Figure 4 and Figure 5, which correspond to sensing structures with varying stacking numbers of graphene and MoS_2_ monolayers, respectively. Notably, monolayer MoS_2_ exhibits stronger light absorption than its bulk form, and the total absorption rises with growing layer count in line with the material’s intrinsic absorption coefficient—each single MoS_2_ monolayer absorbs roughly 5% of the incident light intensity. As observed from both figures, increasing the MoS_2_ layer count causes the SPR resonance dips to broaden and become shallower, a variation trend consistent with that observed in graphene-modified structures.

Table 3 further verifies that the resonance angle will shift when more graphene or MoS_2_ monolayers are added to the sensing architecture. Such a shift in the resonance angle, coupled with a widened reflectance curve and an increased minimum reflectance magnitude, as listed in Table 3, matches well with the remarkable SPR signal modulation induced by coating MoS_2_ and graphene overlayers on metallic thin films. These pronounced changes in the SPR spectral shape ultimately led to a decline in sensing sensitivity. Furthermore, Verma and coworkers [23] demonstrated that introducing an additional dielectric interlayer produces an obvious peak in the sensitivity curve with respect to graphene layer quantities. Their parameterized simulations further reveal that the increasing thickness of such dielectric layers shifts the maximum-sensitivity peak toward structures containing fewer graphene layers.

**Figure 6:**
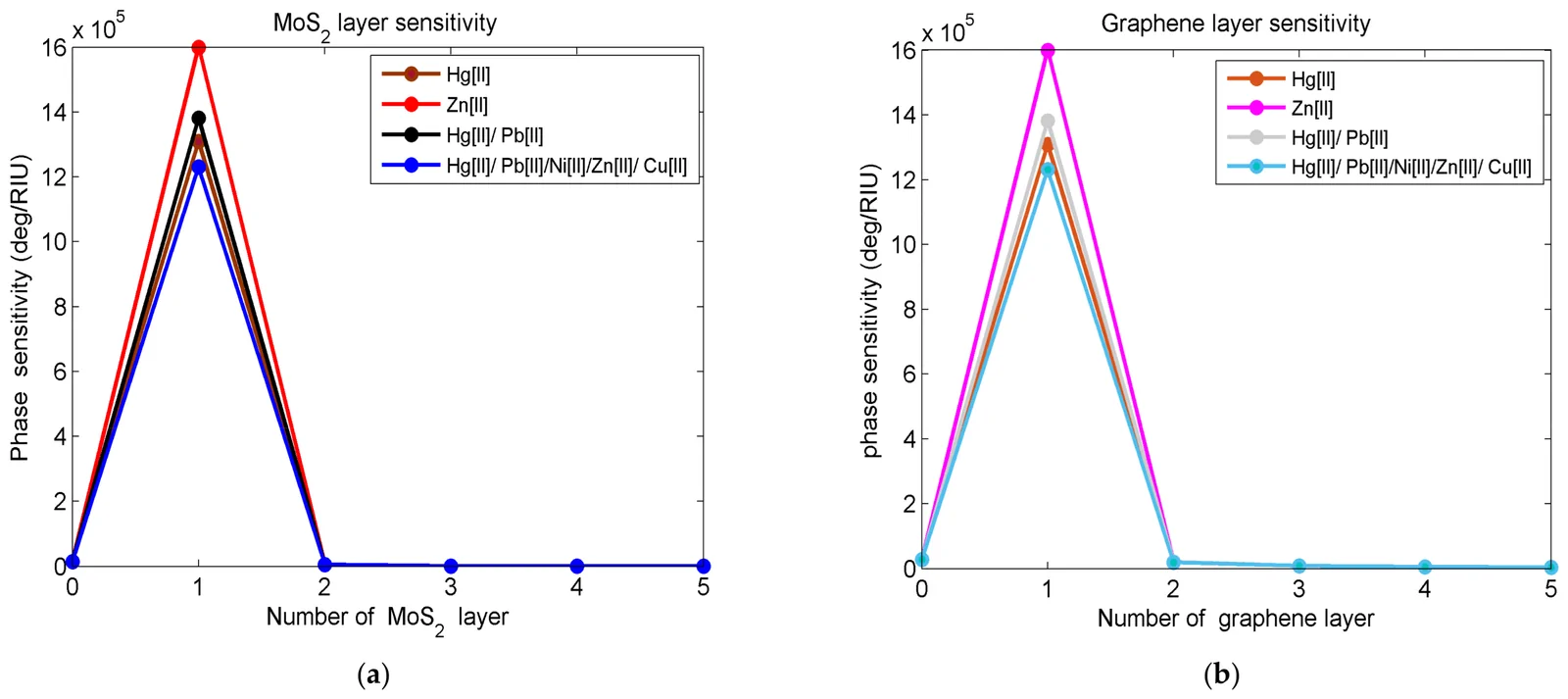
(**a**) Dependence of sensitivity on MoS_2_ layer count (0–5 layers) with monolayer graphene. (**b**) Sensitivity dependence on graphene layer count (0–5 layers) under optimized MoS_2_ layer condition.

Figure 6 presents the variation in phase sensitivity dependent on MoS_2_ and graphene layer quantities when exposed to diverse mixed metal ion analytes. Figure 6a demonstrates that with single–layer graphene deployed, the phase sensitivity for the measurement of diverse mixed metal ions achieves the peak value when only one MoS_2_ layer is adopted. Figure 6b indicates that under optimized thicknesses of Ag and BFO together with the preferred MoS_2_ layer configuration, the sensing curve of the biosensor fails to follow a monotonic trend. The sensitivity rises along with the increasing graphene layer count initially and gradually declines after hitting the peak value. For monolayer graphene, the maximum sensitivities reach 1.308 × 10^6^ deg/RIU, 1.599 × 10^6^ deg/RIU, 1.381 × 10^6^ deg/RIU and 1.230 × 10^6^ deg/RIU for Hg[II], Zn[II], Hg[II]/Pb[II], and Hg[II]/Pb[II]/Ni[II]/Zn[II]/Cu[II] solutions, respectively.

To further verify the superiority of the proposed SPR sensing architecture, this study conducts a performance comparison among four different sensor configurations, and the detailed results are summarized in Table 4. The analytical data in the table reveals that the traditional sensor system merely composed of silver and BFO thin films achieves a maximum phase sensitivity of 1.297 × 10^4^ deg/RIU and a minimum reflectivity of 0.397 in the detection of Hg[II] ions. When graphene is added to the sensing structure, the maximum phase sensitivity is 1.528 × 10^4^ deg/RIU, and the minimum reflectivity is 0.135. Compared with traditional structures, this structure has increased phase sensitivity by 17.81%. By incorporating molybdenum disulfide into the sensing framework, the optimized sensor configuration delivers a substantially improved maximum phase sensitivity of 2.766 × 10^4^ deg/RIU, along with a reduced minimum reflectivity value of 0.017. Compared with traditional structures, this structure has increased phase sensitivity by 113.26%. Under the idealized model, the proposed configuration yields a predicted peak local phase sensitivity of 1.308 × 10^6^ deg/RIU, with the minimum reflectivity dropping to 4.256 × 10^−6^. Compared with the simulated traditional structures, the predicted peak phase sensitivity is approximately two orders of magnitude higher while maintaining the lowest reflectivity. This approximately two-order enhancement is a relative result obtained under the same idealized transfer–matrix assumptions for all four structures: fabrication nonuniformity, interfacial loss, surface roughness, and instrumental phase noise are expected to reduce the experimentally attainable value.

For comparison, Table 5 shows high-performance SPR sensors employing alternative 2D materials, a Ta_2_O_5_ binary oxide, phase-change-assisted critical coupling, and joint optimization strategies. The predicted 1.599 × 10^6^ deg/RIU of the present design is of the same order as recently optimized BlueP/WS_2_/MXene and Cu/ITO/TMDCs phase sensors, with slightly phase sensitivity lower values than other sensing structures, but the structure of these sensors is targeted at different analytes. Therefore, the novelty claimed here is not an absolute sensitivity record; it is the integration of an ultra-thin, low-loss BFO layer with monolayer MoS_2_ and graphene to obtain a critical coupling-enhanced phase response for heavy-metal-ion mixtures.

### 3.3. Spatial Distribution of Electric Field Intensity

**Figure 7:**
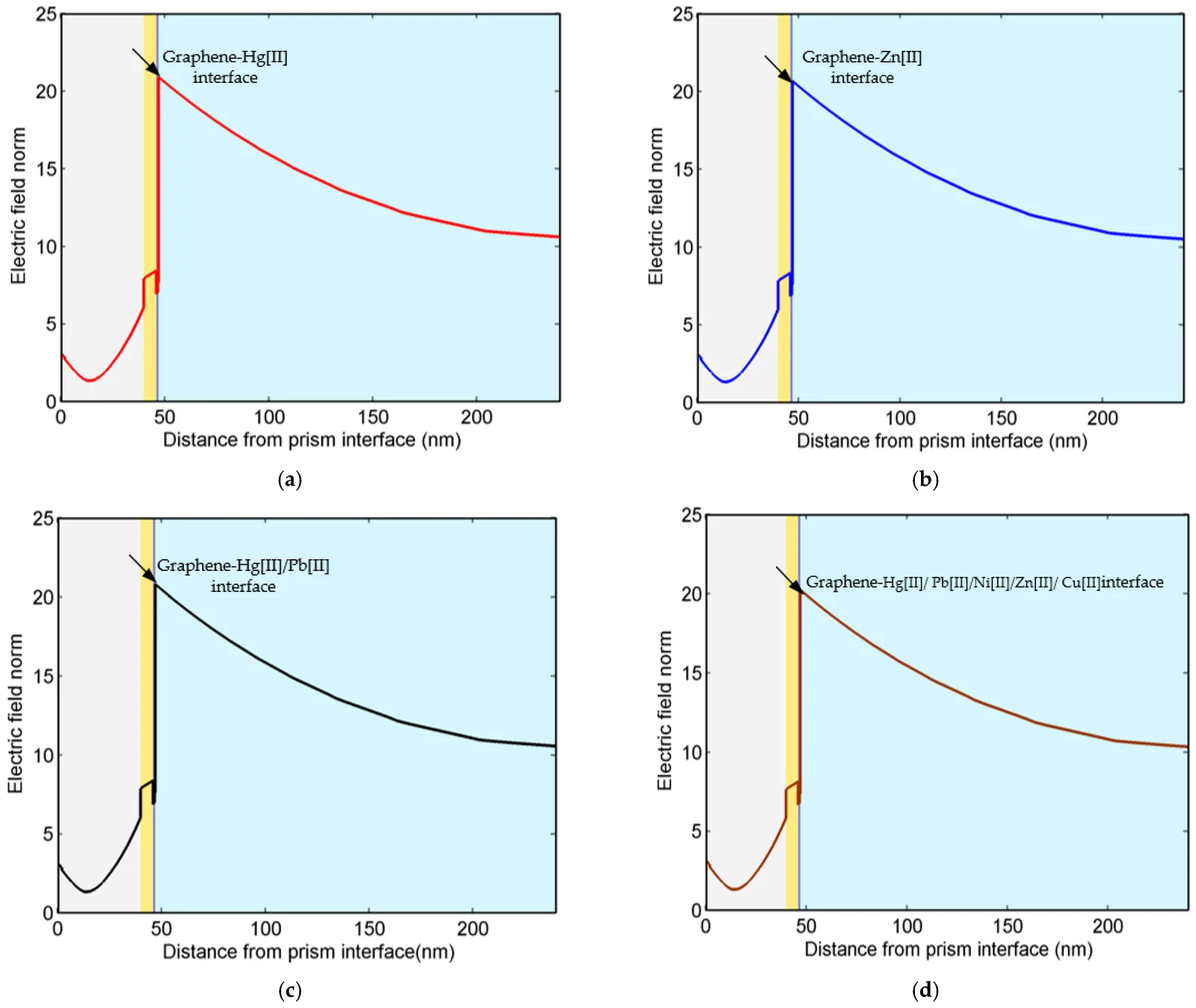
Electric field intensity distribution versus the distance from the prism interface under different heavy metal ion environments: (**a**) Hg[II]; (**b**) Zn[II]; (**c**) Hg[II]/Pb[II]; (**d**) Hg[II]/Pb[II]/Ni[II]/Zn[II]/Cu[II].

The electric field profiles depicted in Figure 7 help further reveal the sensing capabilities achieved by the optimized structure. Subgraphs (a)–(d) demonstrate that covering the conventional SPR sensor surface with MoS_2_ and graphene layers brings about prominent enhancement of electric field strength, indicative of more efficient excitation for surface plasmons (SPs). Variations in the refractive index induced by diverse heavy metal ion concentrations can dramatically alter the electric field intensity at the interface between graphene and the sensing medium. This phenomenon demonstrates that minor fluctuations within the sensing medium are capable of triggering remarkable shifts in the properties of surface propagating waves.

The increase in phase sensitivity is better explained by the complex reflection coefficient than by the multiferroic nature of BFO alone. A 6 nm BFO layer modifies the optical admittance and phase accumulation between Ag and the sensing interface. This adjustment brings the multilayer system close to critical coupling. Under this condition, R_min_ approaches zero, and even a small change in the analyte refractive index can cause a sharp phase shift provides effective admittance control because of its strong real dielectric response. Meanwhile, its imaginary dielectric component is nearly zero at the operating wavelength. It therefore adds little optical absorption. As a result, the resonance dip can become very deep without being substantially broadened by dielectric loss [34,41,44]. At 633 nm, the photon energy is about 1.96 eV. This value is slightly lower than the reported BFO bandgap of 2.09–2.32 eV, supporting the expectation of weak direct interband absorption. The actual absorption may still vary with film stoichiometry and defect concentration. The reported work function of BFO of 4.7 eV may affect the band alignment and the charge transfer at the Ag/BFO/MoS_2_/graphene interfaces. These processes could alter the Fermi level of graphene and the effective optical constants. However, the present model uses fixed refractive indices and does not explicitly describe these electronic interactions. They are therefore not regarded as the direct origin of the simulated improvement. Similarly, the ferroelectric and magnetic properties of BFO may enable external-field control in future devices, but no field-dependent effect is considered in this study. MoS_2_ and graphene also enhance the surface polarizability and shift the maximum evanescent field toward the analyte. This improves the field overlap at the sensing boundary [25,45]. Their optical absorption helps explain why a single monolayer gives the best result. Adding more layers increases damping, broadens the resonance, raises R_min_, and weakens the phase slope, as shown in Figure 4, Figure 5 and Figure 6. Overall, the predicted sensitivity enhancement mainly arises from low-loss admittance matching and the redistribution of the optical field near critical coupling. The electronic and multiferroic characteristics of BFO are more relevant to interface behavior and possible future tuning than to the enhancement calculated in the current model.

### 3.4. Practical Feasibility, Fabrication Tolerances, and Limitations

This work presents a numerical proof of concept. Therefore, the calculated sensitivity should not be treated as an experimentally confirmed detection limit. A possible fabrication process could begin with the deposition of 40 nm Ag on optical-grade BK7 glass by electron-beam evaporation or sputtering. A 6 nm BFO film could then be prepared using low-temperature pulsed-laser deposition or radio–frequency sputtering. Finally, CVD-grown monolayer MoS_2_ and graphene could be transferred onto the structure. Previous studies have demonstrated BFO films as thin as 5 nm and wafer-scale transfer of monolayer MoS_2_ [50,51]. Graphene has also been shown to help preserve the plasmonic properties of Ag [52]. Even so, BFO growth, composition, adhesion, and optical behavior on Ag depend strongly on the fabrication process. Film thickness and optical constants should be examined by ellipsometry or X–ray reflectometry. Atomic force microscopy, Raman mapping, and X-ray photoelectron spectroscopy can be used to evaluate roughness, defects, transfer residues, and interface chemistry. The measured optical parameters should then be incorporated into the transfer-matrix model. Due to the sub nanometer thickness of two-dimensional layers, residues and cracks need special attention [53].

The proposed structure is highly sensitive to fabrication variations because its performance depends on near-critical coupling, with R_min_ = 4.256 × 10^−6^. As shown in Figure 3, increasing the BFO thickness from 6 to 10 nm lowers the sensitivity from 1.308 × 10^6^ to 2.109 × 10^6^ deg/RIU. Changes in the Ag thickness also have a strong influence. Surface roughness in the metal layer can broaden the SPR response [54]. In critically coupled systems, even a 0.2 nm deviation in metal thickness has been predicted to reduce phase sensitivity by more than one order of magnitude [55]. Thus, 40 nm Ag and 6 nm BFO should be viewed as nominal design values rather than established fabrication tolerances. Early experiments should include several samples with thicknesses close to these values. Reflectance and phase measurements can then identify the sample that best approaches critical coupling.

The phase sensitivity reported here represents a local slope and does not directly define the experimental limit of detection. Actual performance will depend on angular and wavelength stability, detector noise, phase-unwrapping errors, temperature–induced refractive–index drift, and differences between fabricated chips. Calibrated phase-sensitive SPR systems have achieved detection levels of 10^−8^ RIU [56]. However, this value cannot be applied directly to the present multilayer configuration. Surface roughness, interface scattering, inaccurate optical constants, and limited angular resolution may increase R_min_ and weaken the phase response. Therefore, the two–order–of-magnitude improvement presented in Table 4 describes a relative numerical advantage under the same ideal conditions. It should not be interpreted as a guaranteed experimental enhancement.

Several factors may reduce device stability over time. Ag can react or oxidize with sulfur through exposed edges, pin holes and cracks. BFO could have interdiffusion or composition changes. The two-dimensional layers could detach from the surface or form cracks or contain transfer residues. A second problem with the use of the sensor in natural water is nonspecific fouling. Graphene can help reduce the corrosion of silver, but it will not fully protect unsealed edges or areas with defects. Low–temperature fabrication, arid storage, edge sealing, a microfluidic sensing aperture, thermal regulation, a reference conduit, and regular blank calibration represent various possible control methodologies. The experimental assessment should encompass measurements of thickness and roughness, calibration of the refractive index, statistics from duplicate chips, controlled mixtures of metal ions, and accelerated aging tests. The empirical thickness tolerance, detection threshold, reproducibility, and operational lifespan must be established through these studies.

## 4. Conclusions

This work develops an innovative Kretschmann–type SPR sensing architecture composed of Ag and BFO thin films integrated with MoS_2_ and graphene to realize superior phase sensitivity. Initially, the thickness parameters of Ag and BFO layers are tuned to attain minimized reflectivity while maintaining satisfactory sensing sensitivity. The influences stemming from varying stacking quantities of MoS_2_ and graphene monolayers on sensor performance are explored under multiple mixed heavy metal ion environments. The configuration utilizing single-layer MoS_2_ and graphene delivers the optimal sensitivity among all tested schemes. When contrasted with conventional SPR architectures, the proposed device adopting optimized Ag and BFO thickness together with monolayer MoS_2_ and graphene achieves a sensitivity enhancement of two orders of magnitude. Subsequent investigations on diverse heavy metal ion samples reveal that the maximum sensitivity of 1.599 × 10^6^ deg/RIU can be achieved for Zn[II] detection. In addition, the electric field distributions related to varied sensing medium refractive indices is also analyzed. The simulated field characteristics further verify the great potential of the designed platform for high-performance metal ion identification. Accordingly, the proposed configuration provides a theoretical design framework for subsequent prototype fabrication and validation. The numerical sensitivity reported here should be interpreted as a design target and theoretical upper bound. Experimental realization will require thickness library screening around the nominal Ag/BFO values, interface and roughness metrology, temperature-referenced phase interrogation, and accelerated stability tests.

## Figures and Tables

**Table 1:** Monolayer thickness and RI parameters of the 2D materials provided at λ=633 nm.

Materials	Monolayer (nm)	RI	Reference
MoS_2_	0.65	5.0805 + 1.1723i	[45]
Graphene	0.34	3.0 + 1.4191i	[45]

**Table 2:** The RI of four types of metal ion mixtures.

Type of Metal Ion Mixtures	RI	Reference
Hg[II]	1.332889	[46]
Zn[II]	1.332842	[46]
Hg[II]/Pb[II]	1.332968	[46]
Hg[II]/Pb[II]/Ni[II]/Zn[II]/Cu[II]	1.333081	[46]

**Table 3:** Five distinct sensing configurations with optimized Ag and BFO film thicknesses under a single-layer MoS_2_ and graphene baseline setup.

Number of MoS_2_ or Graphene Layer	MoS_2_ (with One–Layer Graphene)		Graphene (with One–Layer MoS_2_)	
	$\theta_{res}$ (deg)	R_min_	$\theta_{res}$ (deg)	R_min_
1	78.52	0.097	78.52	0.097
2	81.93	0.379	79.96	0.251
3	81.75	0.645	80.93	0.406
4	81.01	0.773	81.17	0.533
5	80.12	0.835	80.73	0.627

**Table 4:** Phase sensitivity comparison of diverse sensor architectures under optimized Ag and BFO thickness conditions.

Structure No	Layers in the Structure	R_min_	Phase Sensitivity (deg/RIU)
1	Ag–BFO	0.397	1.297 × 10^4^
2	Ag–BFO–MoS_2_	0.017	2.766 × 10^4^
3	Ag–BFO–graphene	0.135	1.528 × 10^4^
4	with all(proposed)	4.256 × 10^−6^	1.308 × 10^6^

**Table 5:** The phase-dependent response of the detector under 633 nm illumination compared against previously reported findings obtained at the identical wavelength.

Reference	Structure	R_min_	Phase Sensitivity (deg/RIU)
[25]	Au–MoS_2_–graphene	/	8.185 × 10^4^
[47]	Ag–Ta_2_O_5_–graphene	/	4.852 × 10^5^
[48]	Cu–ITO–MoSe_2_	/	1.821 × 10^6^
[49]	Ag–BlueP/WS_2_–Ag–MXene	1.271 × 10^−4^	1.866 × 10^6^
This work	Ag-BFO-MoS_2_–graphene	7.498 × 10^−6^	1.599 × 10^6^

## Data Availability

The original contributions presented in this study are included in the article. Further inquiries can be directed to the corresponding author.
